# Inhibition of Bcl-xL sensitizes cells to mitotic blockers, but not mitotic drivers

**DOI:** 10.1098/rsob.160134

**Published:** 2016-08-10

**Authors:** Ailsa Bennett, Olivia Sloss, Caroline Topham, Louisa Nelson, Anthony Tighe, Stephen S. Taylor

**Affiliations:** Manchester Cancer Research Centre, University of Manchester, Wilmslow Road, Manchester M20 4QL, UK

**Keywords:** apoptosis, spindle checkpoint, paclitaxel, taxol, WEHI-539

## Abstract

Cell fate in response to an aberrant mitosis is governed by two competing networks: the spindle assembly checkpoint (SAC) and the intrinsic apoptosis pathway. The mechanistic interplay between these two networks is obscured by functional redundancy and the ability of cells to die either in mitosis or in the subsequent interphase. By coupling time-lapse microscopy with selective pharmacological agents, we systematically probe pro-survival Bcl-xL in response to various mitotic perturbations. Concentration matrices show that BH3-mimetic-mediated inhibition of Bcl-xL synergises with perturbations that induce an SAC-mediated mitotic block, including drugs that dampen microtubule dynamics, and inhibitors targeting kinesins and kinases required for spindle assembly. By contrast, Bcl-xL inhibition does not synergize with drugs which drive cells through an aberrant mitosis by overriding the SAC. This differential effect, which is explained by compensatory Mcl-1 function, provides opportunities for patient stratification and combination treatments in the context of cancer chemotherapy.

## 1. Introduction

Antimitotic agents including the taxanes and vinca alkaloids are used extensively to treat numerous malignancies, including ovarian and breast cancer as well as various leukaemias [1]. Patient responses are, however, unpredictable; some cancers are intrinsically resistant and others acquire resistance. In addition, toxicity to the peripheral nervous system can be problematic [2]. To address these limitations, a raft of second-generation antimitotic agents has been developed, including drugs targeting mitotic kinesins and mitotic kinases [3–7]. Effective clinical use of these novel agents will require the development of predictive biomarkers and patient stratification. This in turn will require an in-depth understanding of the molecular mechanisms by which these drugs kill cancer cells. At present, however, our understanding of the mechanisms by which mitotic dysfunction leads to cell death is still in its infancy.

Traditional antimitotic agents and many second-generation drugs are mitotic blockers [4]. By preventing spindle assembly, these drugs chronically activate the spindle assembly checkpoint leading to a prolonged mitotic arrest which eventually leads to cell death, either directly during mitosis or following slippage back into interphase [8]. Death in mitosis (DiM) and post-mitotic death (PMD) both result from activation of the intrinsic apoptotic machinery, which is regulated by pro-apoptotic and anti-apoptotic members of the Bcl-2 family [9–13]. On the pro-apoptotic side, the BH3-only proteins, Bim, Bid, Bad and Noxa, have been shown to contribute to death in mitosis [14–17]. On the anti-apoptotic side, Bcl-xL and Mcl-1 have been shown to be important mitotic survival factors [18–23]. However, the relative importance of any given Bcl-2 family member is complicated by two issues. First, there is considerable functional overlap among both the pro- and anti-apoptotic factors [12,24–26]. Second, whether these proteins act differently during DiM and PMD is often obscured by population-based approaches that do not distinguish between death in mitosis or following slippage.

Understanding the relative contributions of the various Bcl-2 family members to mitotic cell fate is important from an antimitotic chemotherapy perspective because several pro-survival inhibitors are being evaluated as anti-cancer drugs [27,28]. For example, the BH3 mimetic compound class comprises several molecules that dock into a hydrophobic groove of pro-survival Bcl-2 family proteins, thereby preventing binding of their pro-apoptotic BH3-only partners [29]. Frontrunners in this class include ABT-737 and ABT-263/Navitoclax, related molecules that both inhibit three Bcl-2 family members, namely Bcl-2, Bcl-xL and Bcl-w. Notably, Navitoclax accelerates apoptosis during mitotic arrest induced by either taxol or inhibitors targeting the Eg5 kinesin [21]. In addition, exposing the slippage-prone MDA-MB-231 breast cancer cell line to ABT-737 induces death in mitosis [18]. In these cases, suppressing Bcl-xL using RNAi phenocopied the BH3 mimetic, indicating that Bcl-xL plays an important pro-survival role during a prolonged mitotic arrest.

More recently, novel BH3 mimetics have been developed with enhanced specificity for individual pro-survival Bcl-2 family members, including ABT-199 (which targets Bcl-2 itself) [30], A-1210477 (which targets Mcl-1) [31], and WEHI-539 [32] plus a related compound, A-1155643 [33], both of which target Bcl-xL. A-1155643 enhances the efficacy of docetaxel *in vitro* and in mouse xenograft models [31], further supporting the notion that Bcl-xL resists apoptosis during a prolonged mitotic arrest. However, we recently showed that WEHI-539 induces post-mitotic apoptosis when RKO cells are treated with a low concentration of taxol [12], indicating that Bcl-xL also supports survival following an abnormal mitosis. Therefore, to further explore the role of Bcl-xL in the context of mitotic perturbations, we set out to determine the relative contribution of Bcl-xL to survival following exposure to various antimitotic agents, including mitotic blockers and drivers [4,34]. Moreover, to determine Bcl-xL's role during a prolonged mitotic arrest, following slippage and following an abnormal mitosis, we used single-cell time-lapse imaging to directly correlate mitotic behavior with subsequent cell fate [8].

## 2. Results

### 2.1. Validation of WEHI-539 as an effective Bcl-xL inhibitor

**Figure 1.**
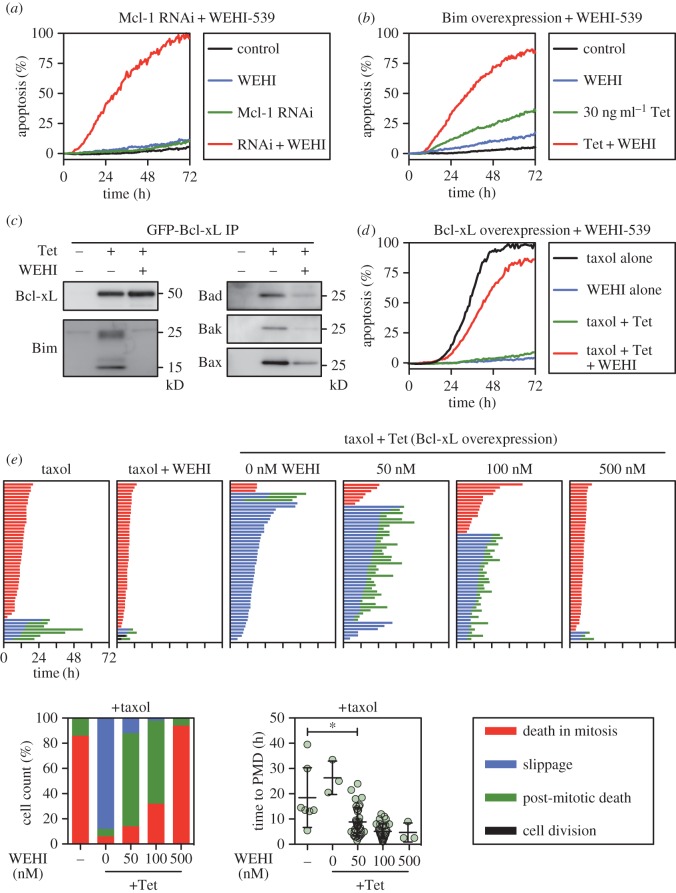
Validation of WEHI-539 as an effective Bcl-xL inhibitor. (*a*) Line graph showing apoptosis induction over a 72 h time course after RKO cells were transfected with siRNAs targeting Mcl-1 for 24 h before exposure to 100 nM WEHI-539. (*b*) Line graph showing apoptosis induction following expression of Bim with 30 ng ml^−1^ tetracycline (Tet) and exposure to 100 nM WEHI-539. (*c*) Immunoblots showing affinity purification of GFP-tagged Bcl-xL induced with 100 ng ml^−1^ tetracycline in the presence and absence of 100 nM WEHI-539, and detection of co-purifying Bim, Bad, Bak and Bax. Note that 100 nM WEHI-539 diminishes binding of pro-apoptotic proteins. (*d*) Line graph showing apoptosis induction by 100 nM taxol, suppression by the induction of Myc-tagged Bcl-xL, and restoration by 100 nM WEHI-539. (*e*) Cell fate profiles of RKO cells exposed to 100 nM taxol following induction of Myc-tagged Bcl-xL and exposure to increasing concentrations of WEHI-539. The number of cells undergoing death in mitosis (red), slippage (blue) and PMD (green) is quantitated in the bar graph. For the cells that undergo PMD, the scatter plot shows the time from mitotic exit to death. *p < 0.05. Zero hours on the fate profiles represents when cells entered mitosis.

WEHI-539 was recently described as a potent and selective Bcl-xL inhibitor [32]. As a BH3 mimetic, it docks into a hydrophobic groove of Bcl-xL, thereby blocking the binding of Bcl-xL's BH3-only partner proteins. To assess WEHI-539 as a research tool in our experimental systems, we first performed four validation experiments. For each we used RKO colon cancer cells in which there is substantial functional overlap between Bcl-xL and Mcl-1: while inhibition of either in isolation has little impact, inhibiting both is sufficient to induce apoptosis in the absence of cytotoxic insult [12] (see the electronic supplementary material, figure S1*a*). To measure apoptosis, we used time-lapse imaging and cell fate profiling in conjunction with a fluorescent caspase 3/7 reporter [8,12]. First, we reasoned that if WEHI-539 is selective for Bcl-xL over Mcl-1, then it should only induce apoptosis in RKO cells in the absence of Mcl-1. Indeed, while WEHI-539 alone was relatively benign up to concentrations of 5 µM (electronic supplementary material, figure S1*a*), following Mcl-1 RNAi, 100 nM was sufficient to induce extensive apoptosis (figure 1*a*; electronic supplementary material, figure S1*a*).

Bcl-xL sequesters multiple BH3-only proteins, including the apoptosis activator Bim which is involved in taxol sensitivity [12,35,36]. Secondly, therefore, we asked whether WEHI-539 exacerbated the ability of a tet-responsive Bim transgene to induce apoptosis (electronic supplementary material, figure S1*b*). Indeed, while induction of Bim with 30 ng ml^−1^ tetracycline only induced moderate apoptosis, 100 nM WEHI-539 substantially enhanced the effect (figure 1*b*). Note that Bim overexpressing cells exposed to WEHI-539 typically died very shortly after mitosis (electronic supplementary material, figure S1*b*), an issue we return to in the Discussion.

Thirdly, we asked whether WEHI-539 blocked the binding of Bcl-xL to its partner BH3-only proteins. Indeed, when we affinity-purified GFP-tagged Bcl-xL in the presence of 100 nM WEHI-539, levels of co-purifying Bim, Bad, Bax and Bak were all reduced (figure 1*c*; electronic supplementary material, figure S1*c*). And finally, because overexpression of a tet-responsive Bcl-xL transgene potently resists taxol-induced apoptosis [12], we asked whether WEHI-539 reversed this effect. Indeed, while induction of Myc-tagged Bcl-xL with 100 ng ml^−1^ tetracycline completely blocked taxol-induced apoptosis, this was reversed by 100 nM WEHI-539 (figure 1*d*; electronic supplementary material, figure S1*d*). Thus, these observations confirm that WEHI-539 behaves as one would expect for a potent and selective Bcl-xL inhibitor.

Because we previously showed that Bcl-xL overexpression resists apoptosis both in mitosis and following slippage [12], we were interested to determine whether the effect of WEHI-539 in the context of Bcl-xL overexpression was via restoration of DiM or PMD. Cell fate profiling showed that in the presence of taxol, 86% of RKO cells underwent DiM and only 14% slipped (figure 1*e*). Every cell that slipped then underwent PMD, with an average of 18.5 h between mitotic exit and death. Consistent with our prior observations [12], overexpression of Bcl-xL dramatically shifted the balance from DiM to slippage, with 94% undergoing slippage. Of these, only 6% underwent PMD, with an average onset time of 26 h. Interestingly, while 50 and 100 nM WEHI-539 only marginally reversed the balance back towards DiM, these concentrations had a major impact on PMD, increasing both the frequency and accelerating its onset (figure 1*e*). By contrast, 500 nM WEHI-539 completely shifted the balance back in favour of DiM. Thus, these observations confirm that Bcl-xL is a potent pro-survival factor both during a prolonged mitotic arrest and following slippage.

### 2.2. WEHI-539 sensitizes cells to microtubule-binding agents

**Figure 2.**
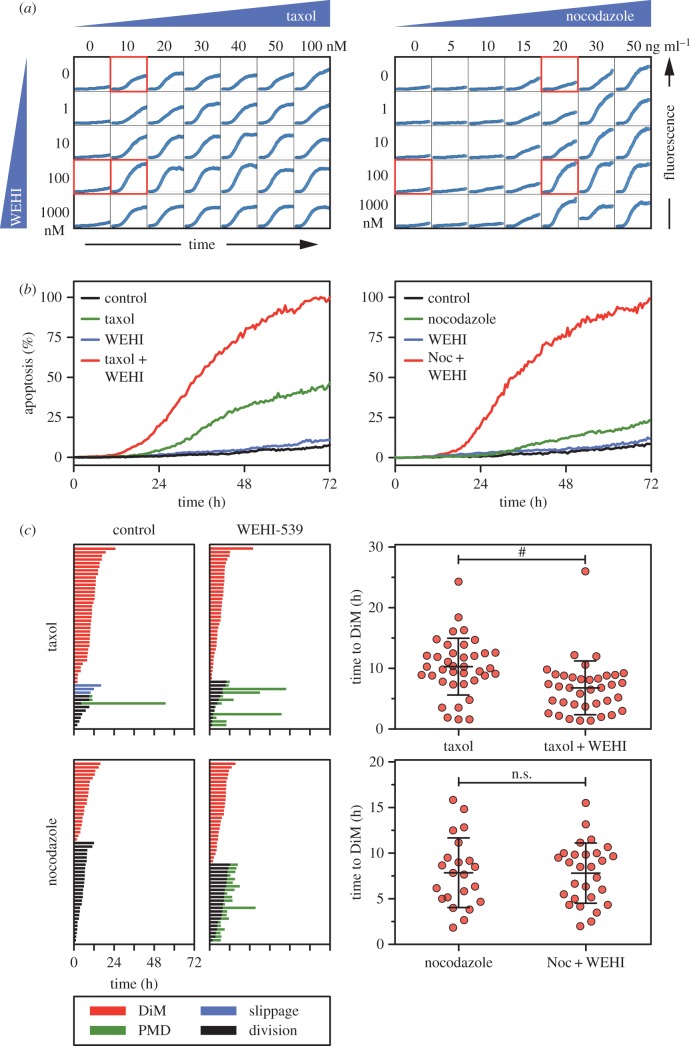
WEHI-539 sensitizes cells to microtubule-binding agents*.* (*a*) Concentration matrices showing apoptosis induction over a 72 h time course following exposure of RKO cells to either taxol or nocodazole and WEHI-539. (*b*) Line graphs showing apoptosis induction following exposure to 10 nM taxol or 20 ng ml^−1^ nocodazole (Noc) plus 100 nM WEHI-539. (*c*) Cell fate profiles of cells as treated in (*b*). For those cells undergoing death in mitosis, the scatter plots quantitate the time from mitotic entry to cell death. ^#^*p* < 0.0001. Zero hours on the fate profiles represents when cells entered mitosis.

Having validated WEHI-539 as a valuable research tool to probe Bcl-xL function in the context of mitotic cell fate, we set out to determine whether it sensitized cancer cells to various mitotic blockers, initially focusing on the microtubule-binding agents taxol and nocodazole. RKO cells were exposed to a matrix of increasing drug concentrations, then caspase 3/7 activation measured by time-lapse imaging over a 72 h time course (figure 2*a*). As shown above, WEHI-539 alone was relatively benign but, as anticipated, taxol and nocodazole induced apoptosis in dose-dependent manners. Importantly, the matrix approach readily identified concentrations where the combination dramatically enhanced apoptosis. Specifically, while 10 nM taxol and 20 ng ml^−1^ nocodazole only induced apoptosis to 46% and 23% of the maximum, respectively, inclusion of 100 nM WEHI-539 induced near-maximal cell death (figure 2*b*). At these relatively low concentrations of antimitotic drug, while many RKO cells underwent DiM, rather than slipping the remainder eventually completed cell division (figure 2*c*). In taxol, some of the dividers underwent PMD but in nocodazole they all arrested in the next interphase. Interestingly, the mechanism by which WEHI-539 enhanced apoptosis differed between the two mitotic blockers. In taxol, WEHI-539 had two effects, accelerating DiM by approximately 3.5 h and increasing the frequency of PMD (figure 2*c*). By contrast, WEHI-539 did not accelerate DiM in nocodazole-treated cells but rather induced PMD in every cell that divided. Nevertheless, despite these differences, these observations build on prior reports [37], and clearly demonstrate that pharmacological inhibition of Bcl-xL sensitizes cells to microtubule-binding agents.

### 2.3. WEHI-539 sensitizes cells to second-generation mitotic blockers

Like the microtubule-binding agents, several second-generation antimitotic drugs also block mitotic progression by disrupting spindle assembly [3,4]. These include inhibitors targeting mitotic kinesins, such as Eg5 and Cenp-E, and mitotic kinases such as Plk1. We therefore asked whether pharmacological inhibition of Bcl-xL also sensitized cells to agents targeting these mitotic regulators, focusing on the Eg5 inhibitor AZ138 [8], the Plk1 inhibitor BI 2536 [38] and the Cenp-E inhibitor GSK923295 [39]. As above, RKO cells were analysed following exposure to a matrix of increasing drug concentrations, which again readily identified combinatorial concentrations that enhanced apoptosis (electronic supplementary material, figure S2*a*). To define this phenomenon in more detail, we performed cell fate profiling at concentrations where the enhancement of apoptosis was particularly marked.

**Figure 3.**
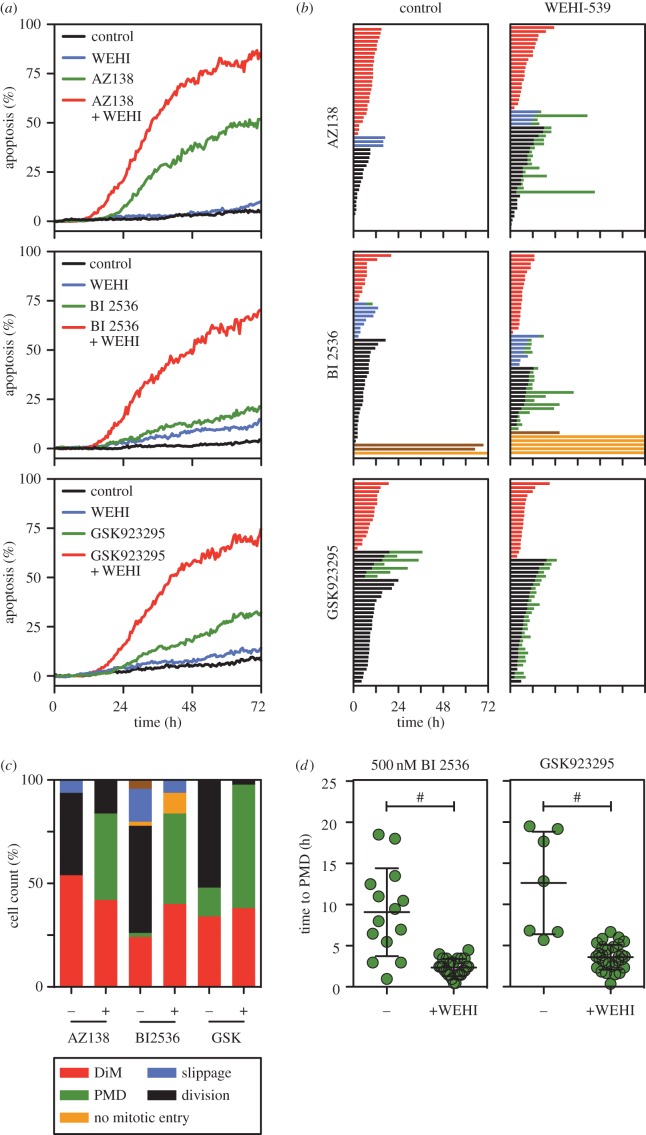
WEHI-539 sensitizes cells to second-generation mitotic blockers*.* (*a*) Line graphs showing apoptosis induction over a 72 h time course following exposure of RKO cells to 200 nM of the Eg5 inhibitor AZ138, 50 nM of the Plk1 inhibitor BI 2536, and 100 nM of the Cenp-E inhibitor GSK923295, plus 100 nM WEHI-539. (*b*) Cell fate profiles of cells treated as in (*a*). (*c*) Bar graph quantitation of (*b*) showing the number of cells undergoing each fate. (*d*) Scatter plot showing the time from mitotic exit to death for the cells that underwent PMD. Note that in the case of the Plk1 inhibitor, the data in panel (*d*) are derived from 500 nM BI 2536. ^#^*p* < 0.0001. Zero hours on the fate profiles represents when cells entered mitosis.

In the case of the Eg5 inhibitor, sensitivity to 200 nM AZ138 was enhanced by 100 nM WEHI-539 (figure 3*a*). Note however that 1 µM AZ138 is typically required to induce a potent mitotic block [8]. Indeed, at 200 nM, while 54% underwent DiM and 6% slipped, 40% eventually divided (figure 3*b*). As above with nocodazole, these dividers never entered another mitosis, suggesting cell cycle arrest. Notably, in the presence of 100 nM WEHI-549, 68% of the dividers underwent apoptosis (figure 3*b*). Thus, Bcl-xL inhibition sensitizes RKO cells to Eg5 inhibition by enhancing PMD rather than DiM. The ability of WEHI-539 to enhance apoptosis was particularly striking in the case of the Plk1 inhibitor BI 2536; whereas 50 nM alone only induced 21% apoptosis, 100 nM WEHI-539 increased this to 70% (figure 3*a*). As with nocodazole and the Eg5 inhibitor, WEHI-539 did not significantly accelerate the time to DiM but rather induced PMD following slippage or cell division (figure 3*b,c*). As above, at the relatively low concentration of 50 nM BI 2536, a substantial proportion of cells eventually divided (figure 3*b*). Therefore, we asked what happened at higher concentrations of BI 2536, which induced a potent cell division block. At 500 nM, the majority of cells underwent DiM or PMD after slippage (electronic supplementary material, figure S2*b*). Of those cells that slipped, 78% of cells underwent PMD after an average of 9.1 h (figure 3*d*). When 100 nM WEHI-539 was included, PMD was accelerated to 2.4 h (figure 3*d*; electronic supplementary material, figure S3*b*). Note that, paradoxically, at BI 2536 concentrations above 500 nM, apoptosis induction was reduced (electronic supplementary material, figure S2*a*), most probably due to suppression of mitotic entry [40]. Nevertheless, as with the Eg5 inhibitor, WEHI-539 sensitizes RKO cells to Plk1 inhibition largely by enhancing PMD. This phenomenon was strikingly apparent with the Cenp-E inhibitor. At 100 nM GSK923295, 66% of cells eventually divided, 21% of which then underwent PMD after an average of 12.6 h (figure 3*b–d*). Upon inclusion of 100 nM WEHI-539, 97% of the dividers underwent PMD after an average 3.6 h. Thus, together with the observations described above, these data demonstrate that inhibiting Bcl-xL can sensitize cells to second-generation mitotic blockers, largely by enhancing post-mitotic cell death.

### 2.4. WEHI-539 only has a minor impact when combined with mitotic drivers

**Figure 4.**
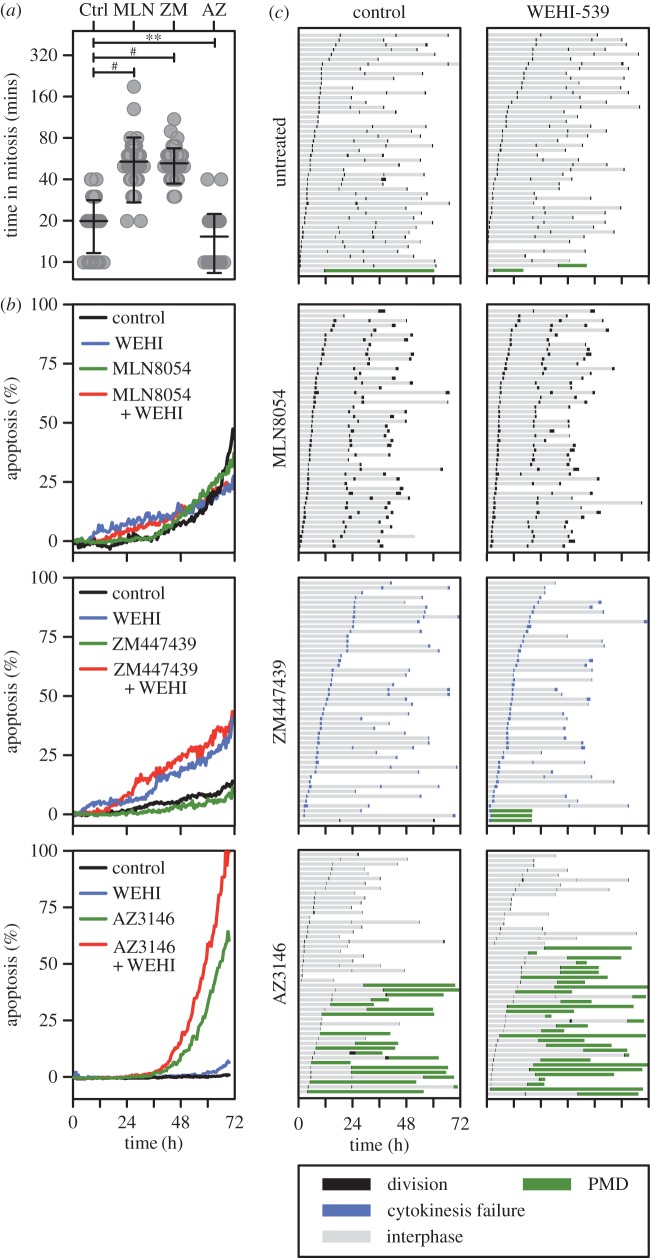
WEHI-539 only has a minor impact when combined with mitotic drivers*.* (*a*) Scatter plot quantitating the time RKO cells spent in mitosis following exposure to 250 nM of the Aurora A inhibitor MLN8054, 2 µM of the Aurora B inhibitor ZM447439 and 2 µM of the Mps1 inhibitor AZ3146. **p < 0.01, ^#^*p* < 0.0001. (*b*) Line graphs showing apoptosis induction following exposure to the drugs indicated in (*a*) plus 100 nM WEHI-539. (*c*) Cell fate profiles of cells treated as in (*b*). Zero hours on the fate profiles represents when imaging started.

In contrast to the microtubule-binding agents, several second-generation antimitotic drugs do not trigger a prolonged mitotic arrest, but rather drive cells through an abnormal division [4]. These include drugs targeting Aurora A, Aurora B and Mps1. To determine whether inhibiting Bcl-xL also sensitized cells to these drugs, we analysed WEHI-539 in combination with the Aurora A inhibitor MLN8054 [41], the Aurora B inhibitor ZM447439 [42] and the Mps1 inhibitor AZ3146 [43]. In isolation, all three drugs induced the expected phenotypes; MLN8054 induced a transient mitotic delay followed by cell division, ZM447439 induced a transient delay followed by cytokinesis failure and AZ3146 accelerated mitotic exit (figure 4*a*,*c*). While inhibiting Aurora A or Aurora B alone was not sufficient to induce apoptosis, at least during the 72 h time course, the Mps1 inhibitor induced PMD in 38% of cells (figure 4*c*). Interestingly, the concentration matrices failed to identify combinations where WEHI-539 significantly enhanced apoptosis (not shown). Even at relatively high concentrations of mitotic driver, WEHI-539 had little additional effect especially in the case of the two Aurora kinase inhibitors (figure 4*b*,*c*). WEHI-539 did have a minor effect when combined with Mps1 inhibitor, increasing PMD to 62% and accelerating its onset by 4 h, although this latter difference was not statistically significant (data not shown). Thus, while pharmacological inhibition of Bcl-xL convincingly sensitizes cells to mitotic blockers, it only has a minor effect when combined with mitotic drivers.

To explore this notion further, we used the concentration matrices to perform a Bliss independence analysis [44,45], and represented the data as heat maps with positive Bliss excess values coloured green and negative values red (electronic supplementary material, figure S3). The heat maps for the microtubule toxins AZ138, BI 2536 and GSK923295 are characterized by patches of green (i.e. positive Bliss excess scores). By contrast, the heat maps for MLN8054, ZM447439 and AZ3146 are dominated by negative values, further supporting the notion that inhibiting Bcl-xL has little impact in the context of a mitotic driver. Accordingly, the mitotic blockers all returned high Bliss sum values, while MLN8054 and ZM447439 returned negative Bliss sums (electronic supplementary material, figure S3*b*). AZ3146 gave a positive albeit low Bliss sum, consistent with the observation that it enhances PMD to some extent (figure 4*c*). Thus, this analysis confirms the notion that while pharmacological inhibition of Bcl-xL sensitizes cells to mitotic blockers, this effect is considerably less pronounced when combined with mitotic drivers.

### 2.5. Overexpression of Mcl-1 suppresses WEHI-539-induced post-mitotic death

To rationalize the differential effect Bcl-xL inhibition had on mitotic blockers versus mitotic drivers, we turned our attention to Mcl-1, a pro-survival factor that is degraded during a prolonged mitotic arrest [46–49] (see the electronic supplementary material, figure S4*b*). We reasoned that if both Mcl-1 and Bcl-xL promote post-mitotic survival, then degradation of Mcl-1 during a prolonged mitotic arrest could account for why cells treated with mitotic blockers become critically dependent on Bcl-xL. Indeed, while Mcl-1 was largely depleted during a nocodazole arrest, its levels remained high in the presence of ZM447439 (electronic supplementary material, figure S4*b*). Therefore, to test this hypothesis further, we modulated Mcl-1 levels in RKO cells and asked whether this altered their sensitivity to combinations of antimitotic agents plus WEHI-539.

**Figure 5.**
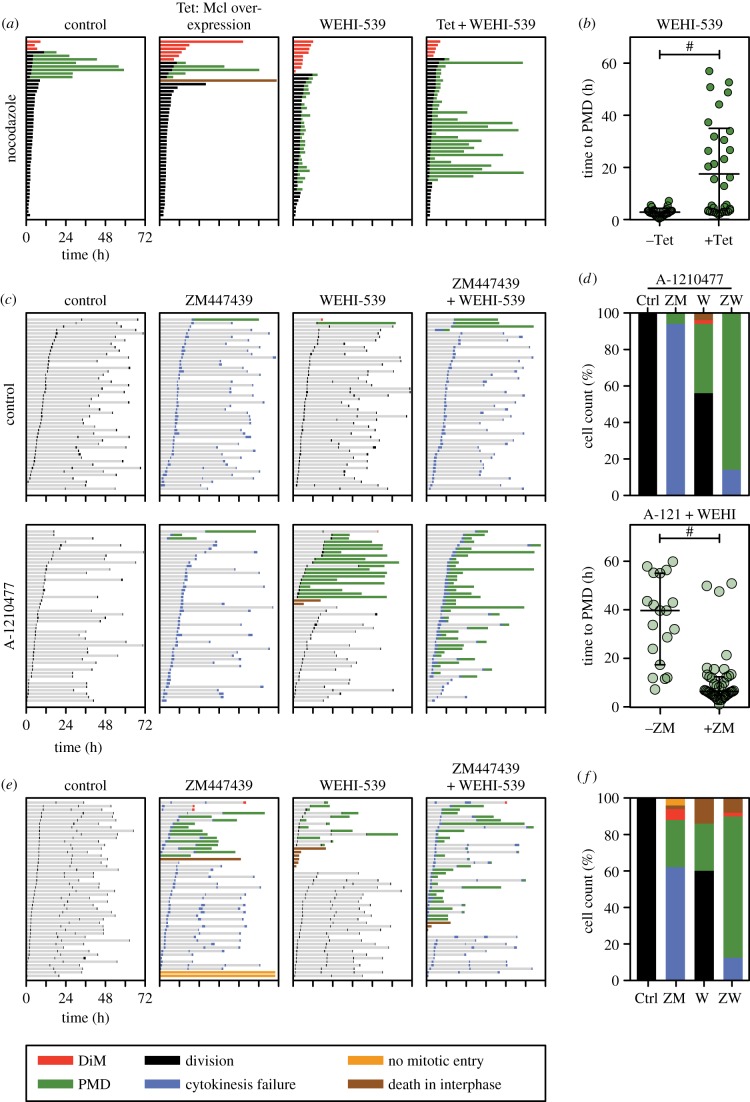
Inhibition of Mcl-1 sensitizes WEHI-539-treated cells to a mitotic driver. (*a*) Cell fate profiles of RKO cells treated with 20 ng ml^−1^ nocodazole and 100 nM WEHI-539 following induction of GFP-tagged Mcl-1 with 1 µg ml^−1^ tetracycline (Tet). (*b*) Scatter plot showing the time from mitotic exit to death for WEHI-539-treated cells in (*a*) that underwent PMD in the presence or the absence of overexpressed Mcl-1. ^#^*p* < 0.0001. (*c*) Cell fate profiles of RKO cells exposed to 2 µM of the Aurora B inhibitor ZM447439, 100 nM WEHI-539 and 2 µM of the Mcl-1 inhibitor A-1210477. (*d*) Bar graph quantitation of (*c*) showing the number of cells undergoing each fate, and scatter plot quantitating the time from mitotic exit to death for the cells that underwent PMD. ^#^*p* < 0.0001. (*e*) Cell fate profiles of DLD-1 cells exposed to 2 µM ZM447439 and 100 nM WEHI-539. (*f*) Bar graph quantitation of (*e*) showing the number of cells undergoing each fate. In panel (*a*), zero hours on the fate profiles represents when cells entered mitosis, while in panels (*c*) and (*e*) it corresponds to when imaging started.

First, we generated an RKO cell line expressing a tet-inducible GFP-tagged Mcl-1 transgene in order to overexpress Mcl-1 (electronic supplementary material, figure S4*a*). When uninduced control cells were exposed to 20 ng ml^−1^ of nocodazole, the vast majority of cells arrested for an average of 4 h then divided (figure 5*a*). Of these, only 17% underwent PMD after an average of 32 h. Consistent with observations shown in figure 2*c*, 100 nM WEHI-539 induced rapid PMD in the vast majority of dividers (figure 5*a*). Overexpression of Mcl-1 only had a minor impact in the absence of WEHI-539, prolonging time to death in the few cells that underwent DiM. However, Mcl-1 overexpression had a significant impact in the presence of WEHI-539, substantially prolonging the onset of PMD in a subset of the cells, extending the average time to PMD from 2.8 to 17.5 h (figure 5*b*). Thus, elevating Mcl-1 suppresses post-mitotic sensitivity to WEHI-539, consistent with the notion that a prolonged mitotic arrest sensitizes cells to Bcl-xL inhibitors by degrading Mcl-1.

### 2.6. Inhibition of Mcl-1 sensitizes cells to WEHI-539 plus an Aurora B inhibitor

Next, we suppressed Mcl-1 by RNAi and asked whether this sensitized Bcl-xL-inhibited cells to a mitotic driver. Consistent with the data in figure 4*c*, adding WEHI-539 to the Aurora B inhibitor ZM447439 had little effect. However, when Mcl-1 was suppressed by RNAi, apoptosis was induced in the majority of cells, either during mitosis or following cell division failure (electronic supplementary material, figure S4*c*). However, this experiment is complicated because co-inhibition of Mcl-1 and Bcl-xL in RKO cells is sufficient to induce apoptosis even in the absence of extrinsic insult [12] (see the electronic supplementary material, figure S1*a*). Indeed, despite reducing the concentration of siRNAs targeting Mcl-1 to 25 nM, when combined with 100 nM WEHI-539, the vast majority of cells died, either in mitosis or shortly thereafter (electronic supplementary material, figure S4*c*). In fact, the addition of the Aurora B inhibitor actually conferred protection, reducing the number of cells dying in mitosis and delaying PMD, possibly due to suppressing telomere deprotection [12,50] (see Discussion). Therefore, to enable better titration of Mcl-1 function, rather than RNAi we turned to the Mcl-1 inhibitor, A-1210477 [31]. Like WEHI-539, A-1210477 is a BH3-mimetic and inhibits Mcl-1 from binding its pro-apoptotic partners (electronic supplementary material, figure S4*d*). First, we analysed a concentration matrix to identify a combinatorial concentration of WEHI-539 and A-1210477 that induced minimal apoptosis (electronic supplementary material, figure S4*e*). When used in isolation, 100 nM WEHI-539 and 2 µM A-1210477 did not suppress proliferation or induce apoptosis, and in combination only had a minor effect (electronic supplementary material, figure S4*e*). Cell fate profiling confirmed that 2 µM A-1210477 in isolation or in combination with ZM447393 had little effect (figure 5*c*). When combined with WEHI-539, 2 µM A-1210477 induced apoptosis in 44% of cells. However, addition of ZM447439 greatly enhanced this, increasing the apoptotic fraction to 86% and accelerating the average time from mitotic exit to death from 35 h to 10 h (figure 5*c*,*d*). Thus, despite the technical difficulties inherent when suppressing both Mcl-1 and Bcl-xL, these observations show that Mcl-1 promotes post-mitotic survival when Bcl-xL-inhibited cells are driven through an abnormal division. In turn, this supports the hypothesis that RKO cells become dependent on Bcl-xL function after exiting a protracted mitosis due to degradation of Mcl-1 during the delay.

### 2.7. Mcl-1-deficient DLD-1 cells are sensitive to WEHI-539 when combined with ZM447439

If both Mcl-1 and Bcl-xL support post-mitotic survival when cells are exposed to mitotic drivers, then we reasoned that cell lines already deficient for Mcl-1 should be sensitive to the combination of a Bcl-xL inhibitor plus a mitotic driver. To test this, we turned to DLD-1 cells, another colon cancer cell line which, compared with RKO, has a relatively lower level of Mcl-1 [49]. In contrast to RKO, exposing DLD-1 cells to WEHI-539 and ZM447439 separately was sufficient to induce apoptosis in a fraction of cells (figure 5*e*,*f*), consistent with weakened Mcl-1 function. More importantly, however, combining WEHI-539 with ZM447439 induced PMD in 76% of cells. Thus, in a cell line lacking robust Mcl-1 function, post-mitotic survival in the presence of a mitotic driver is dependent on Bcl-xL function.

## 3. Discussion

Bcl-xL has previously been implicated as an important mitotic survival factor [12,18,20–22]. To further refine our understanding of Bcl-xL's role when mitosis is disrupted, we have taken advantage of WEHI-539, a BH3 mimetic that selectively and potently blocks binding of Bcl-xL to its BH3-only pro-apoptotic partner proteins [32]. Using concentration matrices to combine WEHI-539 with a panel of representative antimitotic agents, we have systematically analysed Bcl-xL's pro-survival potential in response to drugs that either block or accelerate mitotic progression. In addition, by using cell fate profiling we were able to differentiate death in mitosis from post-mitotic apoptosis. Our results demonstrate that Bcl-xL sustains survival following perturbations that induce a prolonged mitotic delay. By contrast, Bcl-xL function is less critical when cells are driven through an abnormal mitosis, most probably due to the compensatory action of Mcl-1.

### 3.1. Both Bcl-xL and Mcl-1 promote survival following an aberrant mitosis

During mitosis, transcription and translation are heavily suppressed. Indeed, the regulation of mitotic progression is governed largely by post-translational modifications, in particular protein phosphorylation and ubiquitin-mediated proteolysis [51–53]. Accordingly, the apoptotic proteome is also extensively modified during mitosis; for example, Caspase-9, Bid and Bcl-xL are phosphorylated by mitotic kinases [16,22,54–56]. Pro-apoptotic Bim is ubiquitinated by the anaphase-promoting complex (APC/C), the E3 ubiquitin ligase that also targets Cyclin B1 for degradation [15]. However, the net physiological effect these post-translational modifications have on the apoptotic threshold is unclear. Moreover, how these modifications change during a delayed or abnormal mitosis is also not clear. An exception is pro-survival Mcl-1, with several independent reports showing that it is slowly degraded during a checkpoint-mediated mitotic arrest [46,48,49]. Mcl-1 degradation is proteasome dependent and while several E3 ligases have been implicated, the exact mechanism remains to be delineated [49,57]. Nevertheless, several independent observations support the notion that Mcl-1 degradation serves as a mitotic ‘death timer’ mechanism [14,58]. For example, we recently showed that when mitotic exit is blocked in RKO cells by expression of a non-degradable Cyclin B1 mutant, Mcl-1 RNAi accelerates DiM [49]. Conversely, overexpressing Mcl-1 delays taxol-induced DiM by over 5 h. Modulating Mcl-1 also influences post-mitotic survival. We recently showed that Mcl-1 RNAi in slippage-prone DLD-1 cells increases the frequency of apoptosis after slippage, while overexpressing it delays PMD [49]. Taken together with the functional overlap between Mcl-1 and Bcl-xL [12,24,25], these observations allowed us to formulate a simple hypothesis to account for why pharmacological inhibition of Bcl-xL sensitized cells to mitotic blockers but not mitotic drivers, namely that degradation of Mcl-1 during the prolonged delay induced by a blocker renders cells more dependent on Bcl-xL when they eventually exit mitosis. Our subsequent experiment showing that simultaneous inhibition of Bcl-xL and Mcl-1 accelerates PMD in response to an Aurora B inhibitor supports this hypothesis. In turn, this further supports the notion that both Bcl-xL and Mcl-1 contribute to survival following an aberrant mitosis.

### 3.2. In the absence of robust pro-survival activity, mitosis induces apoptosis

Consistent with their overlapping function, simultaneous inhibition of Bcl-xL and Mcl-1 can induce apoptosis even in the absence of cytotoxic insult. This has been shown previously by others [24], but we noted recently that co-repression of Bcl-xL and Mcl-1 by RNAi causes RKO cells to die either in mitosis or very shortly following mitotic exit [12]. Here, we made a similar observation: in the absence of any additional exogenous stress, Mcl-1 RNAi cells exposed to WEHI-539 died either in mitosis or very shortly after mitotic exit. This does not appear to be an RNAi-related phenomenon; when both Bcl-xL and Mcl-1 were inhibited pharmacologically, using WEHI-539 and A-1210477, apoptosis occurred frequently in mitosis or shortly thereafter (not shown). Moreover, when cells overexpressing Bim were exposed to WEHI-539, the vast majority of cells died shortly after completing mitosis. These observations indicate that in the absence of adequate pro-survival activity, mitosis itself is sufficiently stressful to cause apoptosis. One possible trigger for mitosis-specific stress is telomere deprotection, a phenomenon whereby mitotic Aurora B kinase activity displaces TRF2 from telomeres, giving rise to an exposed telomere that is recognized by DNA damage response pathways [50,59]. Consistent with this notion, we previously showed that overexpression of TRF2 or inhibition of Aurora B suppresses mitotic apoptosis in Bcl-xL/Mcl-1-deficient cells [12]. Here, we confirm this in that inhibition of Aurora B delayed apoptosis in Mcl-1RNAi cells exposed to WEHI-539. However, whether telomere deprotection is sufficient to account for apoptosis in cells with weakened pro-survival function remains to be seen. Indeed, this is a very intriguing phenomenon that warrants further investigation. Interestingly, a prolonged mitotic arrest results in mitophagy, reduction in ATP levels and activation of AMPK, and a metabolic switch from oxidative respiration to glycolysis [11]. One possibility therefore is that mitosis-dependent changes in metabolism and/or mitochondrial function could also contribute to mitotic stress that is sufficient to induce apoptosis in cells with weakened pro-survival function.

### 3.3. Identification of patients likely to benefit from antimitotic/Bcl-xL inhibitor combinations

The taxanes and other antimitotic agents are vitally important chemotherapy agents. Yet exactly how they yield patient benefit remains obscure. Recently, the Mps1 inhibitor NTRC 0066-0 was shown to potentiate the anti-tumour activity of docetaxel in a mouse model of triple-negative breast cancer [60]. Strikingly, however, the Mps1 inhibitor alone had little effect, strongly suggesting that docetaxel's anti-tumour activity is via a mitotic mechanism. Indeed, an analysis of breast cancer biopsies indicates that tumour responses correlate with abnormal mitoses observed shortly after taxol infusion [61]. Here, we show that Bcl-xL is a potent pro-survival factor in this context: over a range of clinically relevant taxol concentrations, WEHI-539 enhanced apoptosis by both accelerating death in mitosis and by elevating the frequency of apoptosis following an abnormal mitosis. These observations support and extend previous reports showing that Navitoclax enhances cell death in response to antimitotic agents [18,21,37,62,63]. Together, these observations make a compelling case for exploring Bcl-xL inhibitors in combination with taxanes in order to enhance antimitotic chemotherapy. However, such combinations will inevitably come with an increased toxicity profile. Indeed, Bcl-xL is a molecular clock in platelets, defining their lifespan [64,65]. Consequently, Bcl-xL inhibitors induce thrombocytopenia [66,67], and while this can be ameliorated clinically, judicious use will be required to open up a useful therapeutic window. Interestingly, ovarian cancers with relatively high Bcl-xL levels are less responsive to taxane-based therapy, and ovarian cancer cell lines with higher Bcl-xL/Mcl-1 ratios showed higher Bliss sum values when treated with Navitoclax and taxol [45]. Taken together with our observations, this suggests that patients whose tumours have high Bcl-xL/Mcl-1 ratios might be good candidates for Bcl-xL inhibitor trials in combination with antimitotic agents. Similarly, early-phase clinical trials evaluating novel mitotic blockers (e.g. those targeting Plk1 and Cenp-E) might also benefit by pre-selecting patients with high Bcl-xL/Mcl-1 ratios and exploring combinations with Bcl-xL inhibitors. Conversely, exploring Bcl-xL inhibitors may be less promising in the context of mitotic drivers unless patients whose tumours have low Mcl-1 levels can be identified.

## 4. Material and methods

### 4.1. Cell lines

Parental RKO and DLD-1 cells, plus RKO Flp-In T-Rex derivatives expressing Bim, Myc-tagged Bcl-xL and GFP-tagged Mcl-1, were as described [12,49,68]. All lines were cultured in DMEM plus 10% fetal calf serum (Life Technologies), 100 U ml^−1^ penicillin, 100 U ml^−1^ streptomycin and 2 mM glutamine (all from Sigma), then maintained at 37°C in a humidified 5% CO_2_ atmosphere. A stable RKO line harbouring a tetracycline-inducible GFP-tagged Bcl-xL was generated as described [69]. In brief, a Bcl-xL cDNA [12] was cloned into a pcDNA5/FRT/TO-based vector (Invitrogen) then co-transfected with pOG44 into Flp-In T-Rex RKO cells using Lipofectamine Plus (Thermofisher). Stable integrants were selected in 400 µg ml^−1^ hygromycin B (Roche) and 8 µg ml^−1^ blasticidin (Melford), colonies pooled and expanded to create an isogenic population. To synchronize cells in S-phase, cells were treated with 2 mM thymidine for 16 h.

### 4.2. Small molecule inhibitors

The following drugs were dissolved in DMSO and stored at −20°C: WEHI-539 [32] (Apexbio); taxol (Sigma); nocodazole (Sigma); AZ138 [8] (AstraZeneca)); AZ3146 [43] (AstraZeneca); BI 2536 [38] (Boehringer Ingelheim); GSK923295 [39,70]; MLN8054 [41] (Millennium Pharmaceuticals); ZM447439 [42] (Tocris); A-1210477 [31] (Medchemexpress). Tetracycline (Sigma) was dissolved in water, stored at −20°C, and used at concentrations indicated in the figure legends. Thymidine (Sigma) was dissolved in PBS at a concentration of 200 mM, and stored short-term at 4°C.

### 4.3. RNAi

For RNAi-mediated inhibition, cells were plated in µclear^®^ 96-well plates (Greiner Bio-One) then transfected with a final concentration of 66 nM siRNA using DharmaFECT 1 transfection reagent (Dharmacon) in Opti-MEM media (Life Technologies). siRNAs were ON-TARGETplus SMARTpools (Dharmacon) as described [12] containing oligonucleotides with the following sequences: Bcl-xL (5′-GGACAGCAUAUCAGAGCUU-3′, 5′-GAAAUGAC­CAGACACUGAC-3′, 5′-CCUACAAGCUUUCCCAGAA-3′, 5′-UUAGUGAUGUGGAAGAGAA-3′), Mcl-1(5′ CGAAGGAAGUAUCGAAUUU-3′, 5′-GAUUAUCUCUCGGUACCUU-3′, 5′-GAAGGUGGCAUCAGGAAUG-3′, 5′-GGUUUGGCAUAUCUAAUAA-3′), non-targeting control (5′- UGGUUUACAUGUCGACUAA-3′, 5′-UGGUUUACAUGUUGUGUGA-3′, 5′-GGUUUACAUGUUUUCUGA-3′, 5′-UGGUUUACAUGUUUUCCUA-3′).

### 4.4. Proliferation and apoptosis assays

To measure apoptosis induction and proliferation and to perform cell fate profiling, 1 × 10^5^ cells were seeded per well in µclear 96-well plates (Greiner Bio-One) and IncuCyte Kinetic Caspase-3/7 Apoptosis Assay Reagent (Essen BioScience) added. Note that this cell-permeable reagent consists of a caspase-3/7 recognition motif (DEVD) coupled to a DNA intercalating dye. Upon cleavage by activated caspase-3/7, the liberated dye binds nuclear DNA and emits green fluorescence [71]. Fluorescence values were then normalized to control wells on the same plate which exhibited maximum apoptosis yielding percentage apoptosis values which were plotted. Note also that to maximize fluorescence detection, DMEM was replaced with Leibovitz's L-15 (Sigma-Aldrich). Cells were then imaged using an IncuCyte ZOOM (Essen BioScience) equipped with a 20× objective and maintained at 37°C in a humidified 5% CO_2_ atmosphere. Phase contrast and fluorescence images with two to four images per well were collected every 10–30 min and IncuCyte ZOOM software used in real-time to measure confluency, as a proxy for proliferation, and apoptosis, respectively. Image sequences were then exported in MPEG-4 format and analysed manually to generate cell fate profiles [8]. IncuCyte ZOOM data and timing data were imported into Prism 6 (GraphPad) for statistical analysis and presentation. Note that zero hours on the fate profiles represent either when cells entered mitosis or when imaging was started; see individual figure legends for details.

### 4.5. Immunoprecipitation

Flp-In T-Rex RKO cells harbouring an inducible GFP-tagged Bcl-xL transgene were seeded in 6-well plates and 100 ng ml^−1^ tetracycline added overnight. 10 × 10^5^ cells were then harvested and lysed in 1 ml of buffer containing 0.1% Triton X-100, 100 mM NaCl, 10 mM Tris pH 7.4, 1 mM EDTA, 1 mM EGTA, 20 mM β-glycerophosphate, 10 mM NaF and protease/phosphatase inhibitors (Roche). The lysate was then clarified by centrifugation at 16 000*g* for 20 min at 4°C. To 1 ml of supernatant, 30 µg of a GST-GFP-nanotrap fusion protein was added [49,72] along with glutathione sepharose beads (Amintra). After incubation at 4°C with rotation for 2 h, beads were harvested by centrifugation and washed five times with lysis buffer. Bound proteins were eluted by boiling in sample buffer (0.35 M Tris pH 6.8, 0.1 g ml^−1^ sodium dodecyl sulfate, 93 mg ml^−1^ dithiothreitol, 30% glycerol, 50 µg ml^−1^ bromophenol blue) then resolved by SDS-PAGE.

### 4.6. Immunoblotting

Following SDS-PAGE, proteins were electroblotted onto Immobilon-P membranes. Following blocking in 5% dried skimmed milk (Marvel) dissolved in TBST (50 mM Tris pH 7.6, 150 mM NaCl, 0.1% Tween-20), membranes were incubated overnight at 4°C with the following primary antibodies diluted in TBST: 54H6 (Rabbit anti-Bcl-xL, 1 : 1000; Cell Signalling Technology), S-19 (Rabbit anti-Mcl-1, Santa Cruz Biotechnology), sheep anti-Tao1 (1 : 3000 [73]), rabbit anti-Bim (1 : 500; BD Biosciences), mouse anti-Bad (1 : 1000; Santa Cruz), rabbit anti-Bid (1 : 1000; Cell Signalling), mouse anti-Bax (1 : 1000; BD BioSciences), mouse anti-Bak (1 : 1000; Calbiochem), 4A6 (mouse anti-Myc tag; 1 : 1000; Millipore) and GFP (Rabbit anti-GFP; 1 : 1000; Cell Signalling). Membranes were then washed three times in TBST and incubated for at least 1 h with appropriate horseradish-peroxidase-conjugated secondary antibodies (Zymed). After washing in TBST, bound secondary antibodies were detected using either EZ-ECL Chemiluminescence Reagent (Biological Industries) or Luminata Forte Western HRP Substrate (Millipore) and a Biospectrum 500 imaging system (UVP).

### 4.7. Statistical methods and Bliss independence analysis

Prism v. 6 (GraphPad) was used for statistical analysis, with non-parametric Mann–Whitney *U*-tests for all figures, where *p < 0.05, **p < 0.01, ***p < 0.001, ^#^*p* < 0.0001, n.s.: *p* > 0.05. Lines on scatterplots show mean and interquartile ranges. Combination synergy of WEHI-539 with antimitotic agents was determined by Bliss independence analyses [44,45]. A Bliss expectation for a combined response (*C*) was calculated by the equation: *C* = (*A* + *B*) − (*A* × *B*), where *A* and *B* are the percentage apoptosis induced by drug A and B at a given dose. The difference between the Bliss expectation and the extent of apoptosis observed is the Bliss excess. Bliss excess scores were then summed across the dose matrix to generate a Bliss sum, where a value equal to zero indicates that the combination is additive, a value greater than zero indicates synergy, while a Bliss sum of less than zero indicates antagonism.

## Supplementary Material

Electronic supplementary material

## Data accessibility

Research materials are available upon request to S.S.T.
