# Supplementary material for: Inhibition of Bcl-xL sensitizes cells to mitotic blockers, but not mitotic drivers
Source: Open Biol. 2016 Aug 10;6(8):160134. doi: 10.1098/rsob.160134 (PMC5008013; doi:10.1098/rsob.160134)
Supplement: Electronic supplementary material [file rsob160134supp1.pdf]

Electronic supplementary material

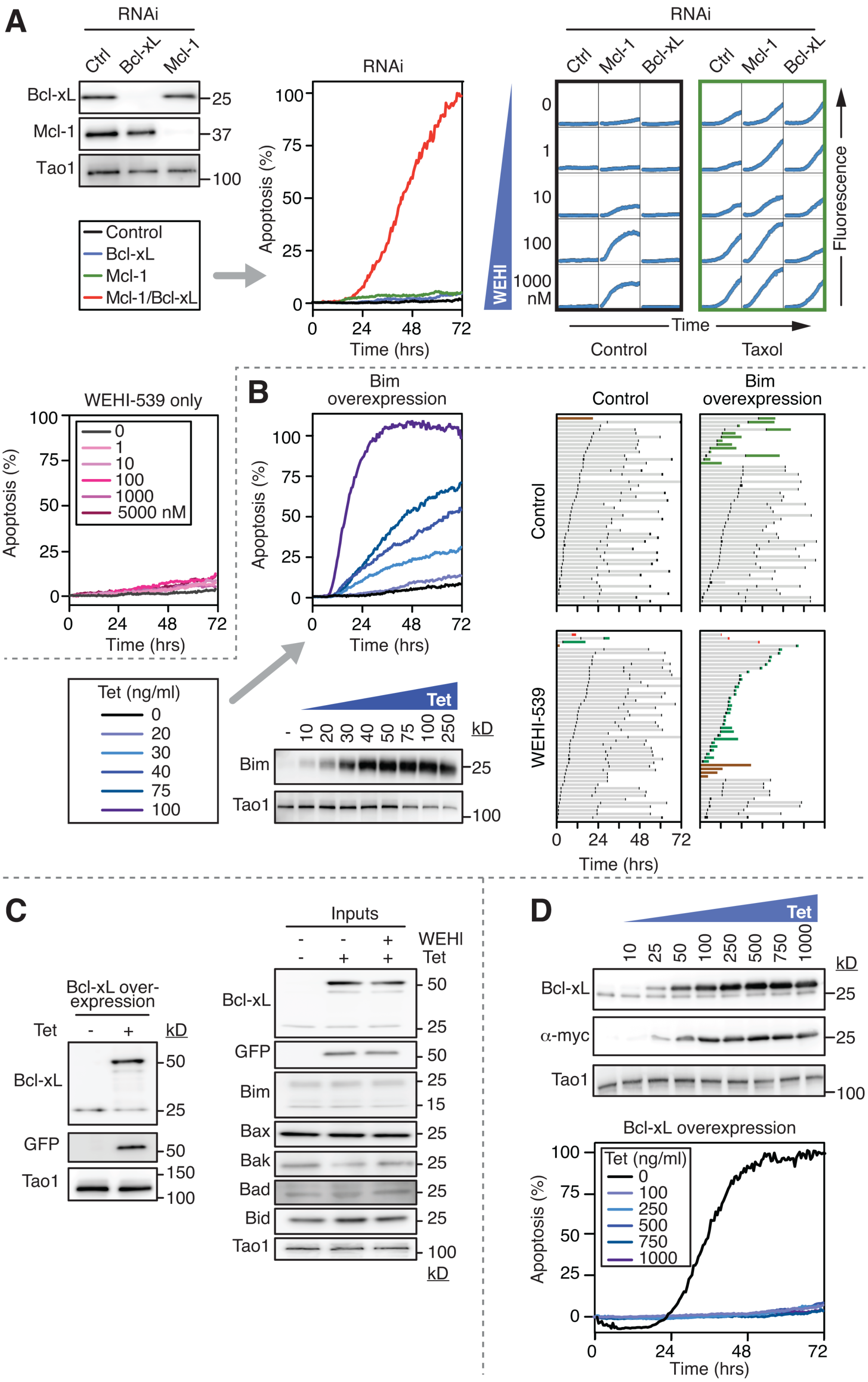

Figure S1 related to Figure 1. Validation of WEHI-539 as an effective Bcl-xL inhibitor. (**A**) Immunoblot showing Mcl-1 and Bcl-xL protein levels following transfection of RKO cells with siRNAs targeting Mcl-1 and Bcl-xL for 24 hours. Line graph showing apoptosis induction over a 72 hour time course following RNAi-mediated inhibition of Mcl-1 and Bcl-xL. Concentration matrix showing apoptosis induction following RNAi and exposure to 100 nM taxol addition. Line graph showing apoptosis induction following exposure to increasing concentrations of WEHI-539. (**B**) Immunoblot, apoptosis line graph and cell fate profiles following tetracycline induced overexpression of Bim. The cell fate profiles are derived from cultures exposed to 30 ng/ml tetracycline and 100 nM WEHI-539. (**C**) Immunoblot showing induction of GFP-tagged Bcl-xL in RKO cells treated with 100 ng/ml tetracycline and the inputs used for affinity purification shown in Fig. 1C. (**D**) Immunoblot and apoptosis line graph following induction of Myc-tagged Bcl-xL with increasing concentrations of tetracycline and exposure to 100 nM taxol. Zero hours on the fate profiles represents when imaging started.

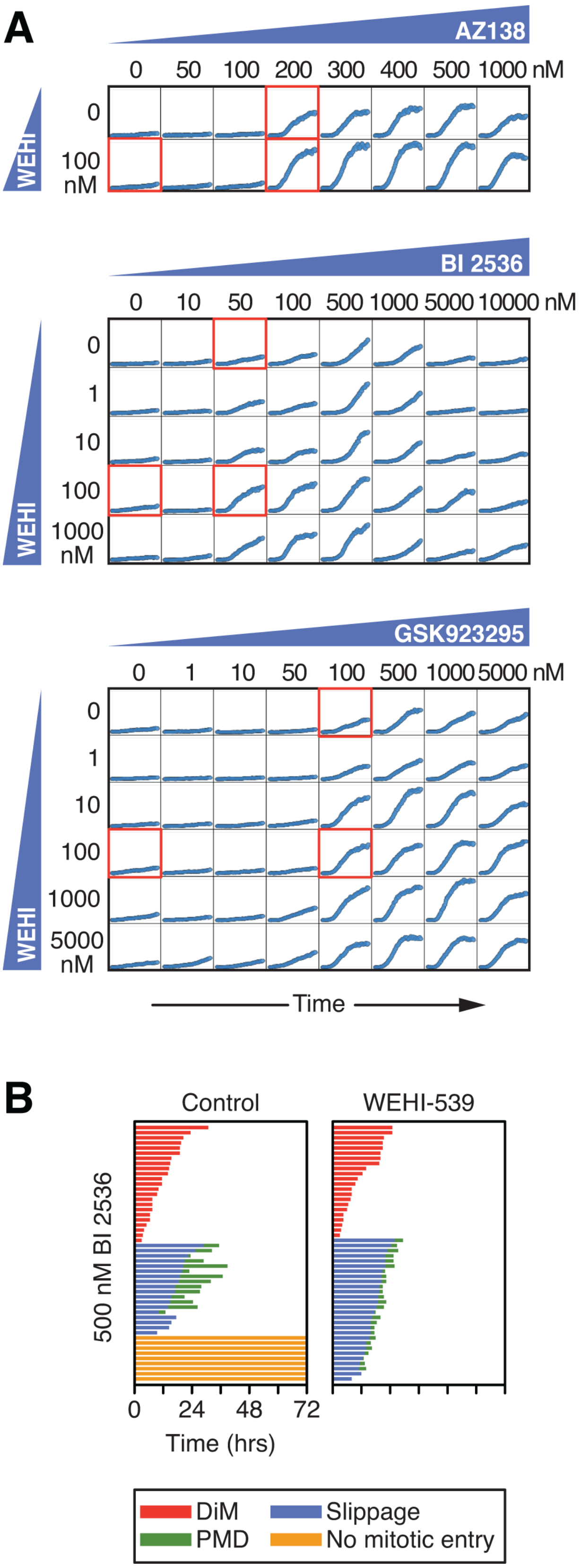

Figure S2 related to Figure 3. WEHI-539 sensitizes cells to second generation mitotic blockers. (**A**) Concentration matrices of RKO cells treated with varying concentrations of the Eg5 inhibitor AZ138, the Plk1 inhibitor BI 2536, and the Cenp-E inhibitor GSK923295, plus WEHI-539 showing apoptosis induction over a 72 hour time course. (**B**) Cell fate profiles of RKO cells treated with 500 nM BI 2536 with and without 100 nM WEHI-539. Zero hours on the fate profiles represents when cells entered mitosis.

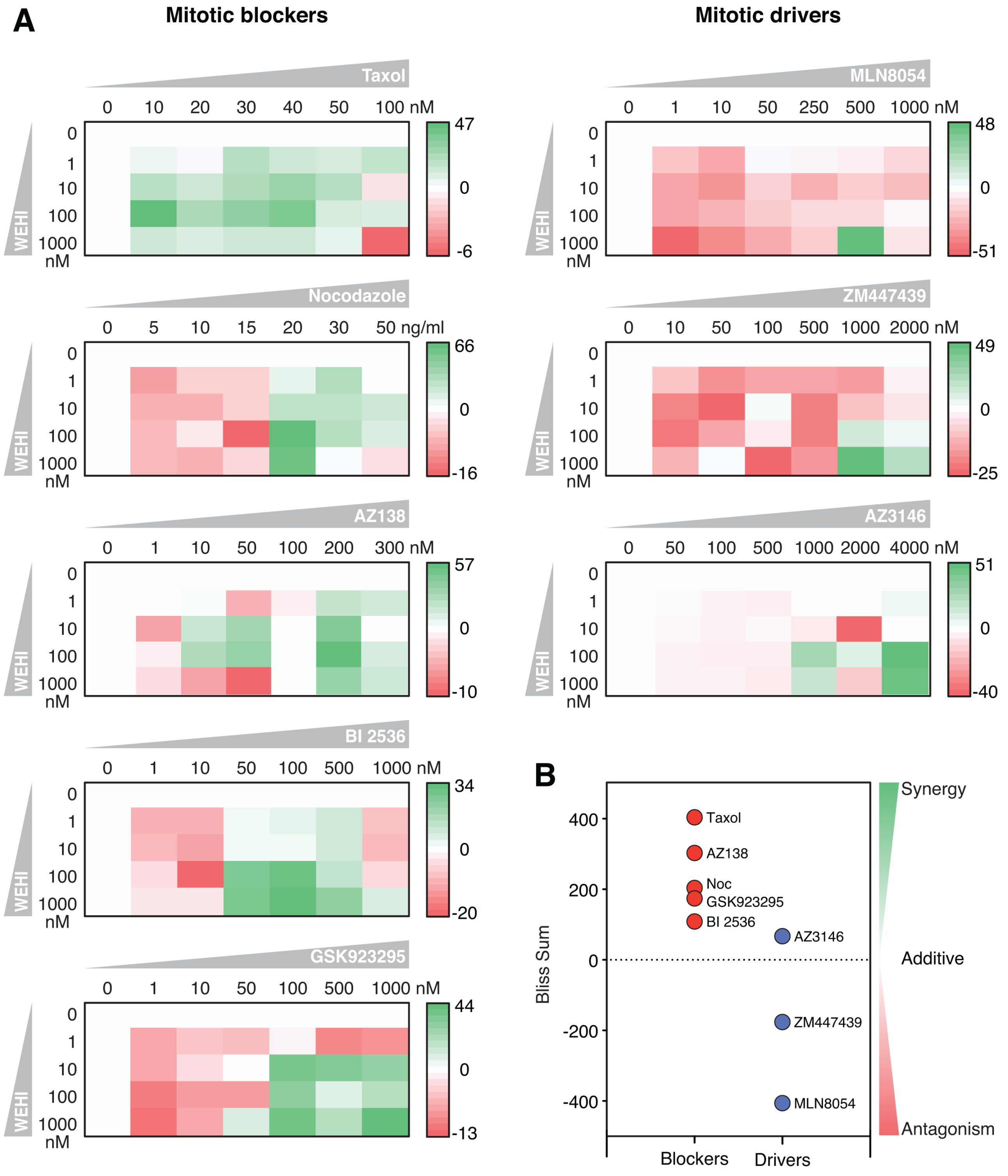

Figure S3 related to Figures 3 and 4. WEHI-539 synergises with mitotic drivers but not mitotic blockers. (**A**) Heat maps showing the Bliss excess values derived from the concentration matrices of RKO cells treated with the drugs indicated. (**B**) Scatter plot showing the Bliss sum values for the mitotic blockers (red) and the mitotic drivers (blue).

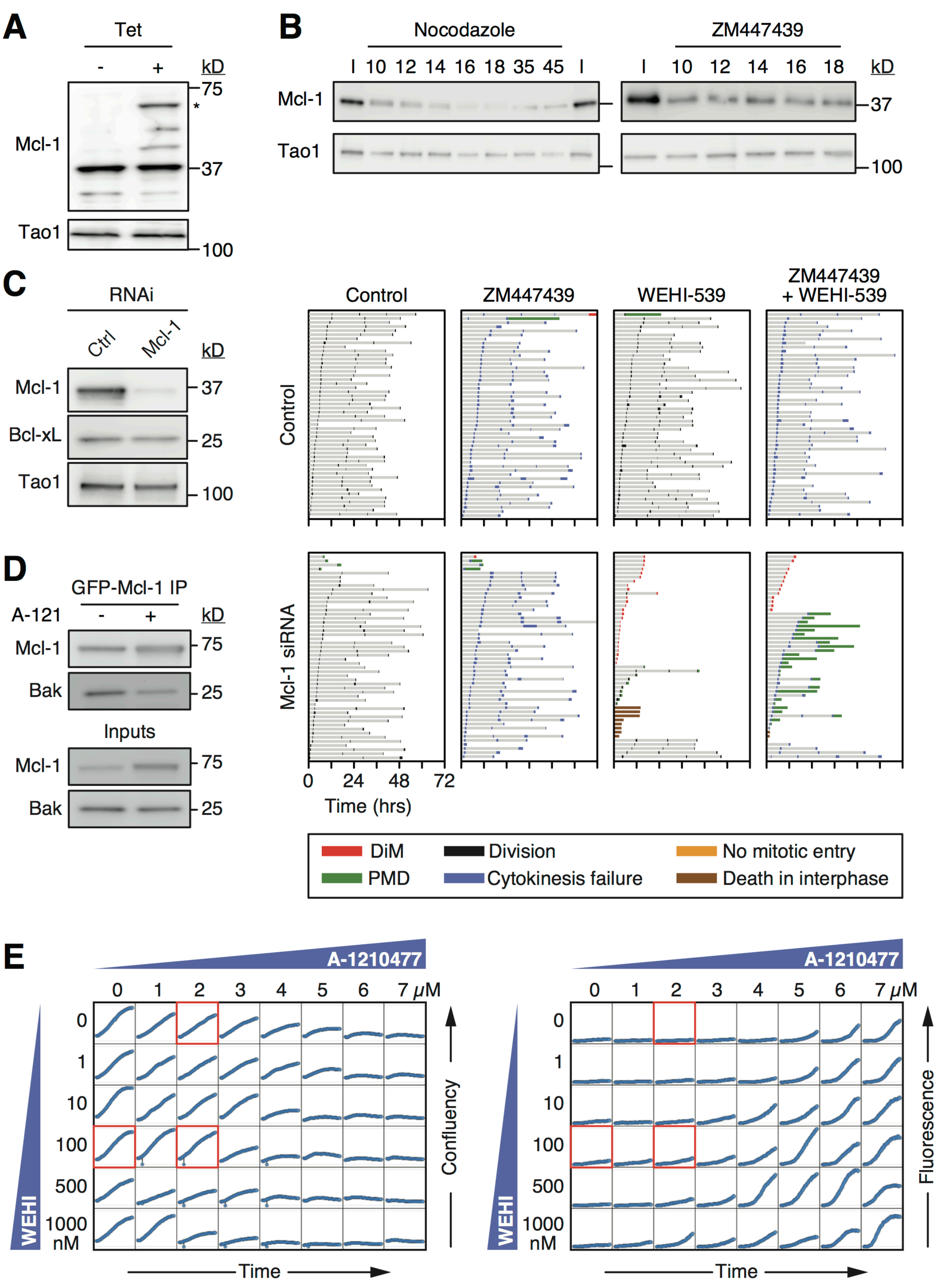

Figure S4 related to Figure 5. Inhibition of Mcl-1 sensitizes WEHI-539-treated cells to a mitotic driver. (**A**) Immunoblot showing induction of GFP-tagged Mcl-1 (marked with *) in RKO cells following induction with 1 μg/mL tetracycline. (**B**) Immunoblot of endogenous Mcl-1 levels in RKO cells following a single thymidine block-and-release into either 20 ng/ml nocodazole or 2 μM ZM447439. Protein samples were harvested at various hours after release as indicated. An interphase sample (I) is shown for comparison. (**C**) Immunoblot and cell fate profiles following transfection of RKO cells with 25 nM siRNA targeting Mcl-1 and exposure to 2 μM ZM447439 and 100 nM WEHI-539. (**D**) Immunoblots showing affinity purification of GFP-tagged Mcl-1 in the presence of 2μM A-1210477, and detection of co-purifying Bak. (**E**) Concentration matrices showing confluency and apoptosis induction of RKO cells over a 72 hour time course following exposure to the Mcl-1 inhibitor A-1210477 and WEHI-539. Zero hours on the fate profiles represents when imaging started.
